# Formation of Si Nanorods and Discrete Nanophases by Axial Diffusion of Si from Substrate into Au and AuPt Nanoalloy Nanorods

**DOI:** 10.3390/nano10010068

**Published:** 2019-12-27

**Authors:** Nele Berger, Ayoub Laghrissi, Yee Yan Tay, Thirumany Sritharan, Jacek Fiutowski, Horst-Günter Rubahn, Mohammed Es-Souni

**Affiliations:** 1 Institute for Materials & Surface Technology, Kiel University of Applied Sciences, 24149 Kiel, Germany; Nele.berger@gmx.net (N.B.); Ayoub.laghrissi@fh-kiel.de (A.L.); 2 School of Materials Science and Engineering, Nanyang Technological University, Singapore 639798, Singapore; yytay@ntu.edu.sg; 3 Mads Clausen Institute, University of Southern Denmark, NanoSYD, Alsion 2, 6400 Sønderborg, Denmark; fiutowski@mci.sdu.dk (J.F.); rubahn@mci.sdu.dk (H.-G.R.)

**Keywords:** 1D-nanostructures, Au nanorods, Pt nanorods, AuPt nanoalloy, diffusion, platinum silicide, Si nanorods, Au nanoparticles, AAO thin films, electrodeposition in AAO

## Abstract

Interdiffusion between Si substrate and nanorod arrays of Au, Pt, and AuPt nanoalloys is investigated at temperatures lower than the AuSi eutectic temperature. When the nanorod is pure Au, Si diffusion from the substrate is very rapid. Au atoms are completely replaced by Si, converting the nanostructure into one of Si nanorod arrays. Au is diffused out to the substrate. The Au nanorod arrays on Si are unstable. When the nanorod is pure Pt, however, no diffusion of Si into the nanorod or any silicide formation is observed. The Pt nanorods are stable on Si substrate. When the nanorods are an alloy of AuPt, interesting interactions occur. Si diffusion into the nanorods is rapid but the diffusing Si readily reacts with Pt forming PtSi while Au diffuses out to the substrate. After annealing, nanophases of Au, Pt, PtSi, and Si may be present within the nanorods. When the Pt content of the alloy is low (12 at%) all Pt atoms are converted to silicide and the extra Si atoms remain in elemental form, particularly near the tip of the nanorods. Hence, the presence of Au accelerates Si diffusion and the ensuing reaction to form PtSi, a phenomenon absents in pure Pt nanorods. When the Au content of the alloy is low, the Si diffusion would cease when all Au atoms have diffused out of the nanorod, thereby arresting the silicide formation resulting in excess Pt in elemental form within the nanorod. This is a technique of making Si nanorods with and without embedded PtSi nanophase consisting of heterojunctions which could have unique properties.

## 1. Introduction

It has been known for a long time that Au/Si interfaces promote intermixing and migration of Si atoms in Au and vice versa at room temperature [1]. It is a unique feature of Au/Si junctions that even at room temperature Si atoms can outdiffuse to the surface of several hundred Å thick gold layers [2]. The intermixing of Au and Si even promotes the crystallization of Si at temperatures below 250 °C in comparison to the crystallization temperature of 550 °C known for pure Si [3]. Further, AuSi is a well-known eutectic system with a eutectic temperature of 363 °C at a Si composition of 19 at% [1], and this has been technologically exploited for low-temperature wafer-to-wafer bonding for micro-electromechanical applications [4]. In AuSi thin film heterostructures, Hiraki et al. [5] demonstrated the migration of Si atoms to the Au surface at a temperature as low as 150 °C where they reacted with the ambient to form a SiO_2_ scale. When the Au layer was replaced by Pt the formation of platinum silicide was observed at low temperatures, down to 300 °C, which is well below the eutectic temperature. Additionally, there is a lot of discussion in the literature regarding the influence of Au on the formation of platinum silicide in AuPt heterostructures [5,6,7]. Kanamori et al. [6] reported that Au promotes the formation of platinum silicide in a SiTiPtAu heterostructure. However, Ti could also serve as a barrier to restrain this reaction if its thickness is sufficiently large. An Au layer between the Si and Pt layers seemed to boost the formation of PtSi at a temperature between 225 °C and 350 °C, while the formation rate of Pt_2_Si was not affected [7]. These studies were motivated by the industrial relevance of Au and Pt ohmic contacts with Si. More recently, the formation of silicides between a Si nanowire (NW) and metal contact pads was addressed [8,9]. Lin et al. [8] described the formation of single crystal PtSi NW when a heterostructure of Si NW and lithographically defined Pt contact pads were annealed at 520 °C for 60 s. They also showed that the PtSi NW grows with a definite epitaxial relationship with Si. When the contact pads consisted of two different metals, e.g., Pt and Ni as in the work of Wu et al. [9], in situ growth of dual silicide NWs, namely Pt_2_Si and Ni_2_Si, was observed during annealing at 650 °C, with the reaction fronts starting from each metal contact. These results are interesting as they suggest preferential diffusion of the metal towards the Si NW.

Au and Pt nanoparticles (NPs) are also very well known for catalyzing Si NW formation in the vapor–liquid–solid (VLS) process that is claimed to generate a high density of single crystalline NWs [10,11,12,13,14]. Si NWs are interesting as they show a range of interesting properties that make them potential candidates for a number of applications spanning solar energy harvesting to nanoelectronics [15,16].

Platinum silicide, PtSi, is an equiatomic intermediate compound that spontaneously forms from the melt or via solid state reaction between Pt and Si [17]. It is a line compound with a melting temperature of 1230 °C, has a rhombohedral structure belonging to space group Pnma, and is a well-known semiconductor and a Schottky barrier material with good thermal and mechanical stability. It can be used in a variety of applications including infrared detection, thermal imaging, ohmic and Schottky contacts [18,19,20], and Atomic Force Microscopy tips [21].

Inspired by the works cited above, we embarked on this project to investigate the diffusion of Si when the nanorods (NRs) are fully metallic and are supported on substrates containing Si. We chose elemental Au, elemental Pt, and AuPt nanoalloys to form the NRs on Si substrate. This system was used as the platform for this investigation. NR arrays of these materials were electrochemically grown in a thin anodic aluminum oxide (AAO) template supported on a SiTiAuTi heterostructure substrate. The AuPt nanoalloys NRs were grown using mixtures of Au and Pt electrolytes of different molar ratios. The results showed that these material systems behaved in a complex manner where not only thermodynamics but also kinetics governed the ensuing reactions depending on the chemistry of the metal NR array. This work also demonstrated the possibility of growing Si NRs starting from Au NRs on a Si substrate by solid-state diffusion in contrast to the well-known VLS growth process.

## 2. Materials and Methods

The following chemicals were used as purchased: Oxalic acid dihydrate 99% (Roth, Karlsruhe, Germany), phosphoric acid 88% (Roth, Karlsruhe, Germany), sulfuric acid 96% (Roth, Karlsruhe, Germany), nitric acid ≥ 65% (Fluka, Neu Ulm, Germany), hydrogen hexachloroplatinate (IV) solution (Fluka, Neu Ulm, Germany), gold (III) chloride trihydrate (Sigma Aldrich, Germany), sodium hydroxide (Roth, Karlsruhe, Germany), ammonium hydrogen difluoride (Fluka, Neu Ulm, Germany), and ethanol. Deionized water was used to prepare aqueous solutions. Silicon (100) wafers were used as substrates.

All Si wafers were cleaned and stripped of the native oxide layers by immersion in sulfuric acid 96% for 1 h and nitric acid ≥ 65% for 2 h, both in an ultrasonic bath, followed by rinsing in 1% hydrofluoric acid (HF), before use. The NR arrays were prepared using substrate-supported AAO thin film templates, whose preparation was described in detail in previous works [22,23,24,25,26]. Briefly, 100 Si wafers were first coated with a thin titanium adhesion layer (6 nm) followed by a gold underlayer (15 nm) via sputtering. On top of this heterostructure a 500 nm aluminum layer was electron beam evaporated. Anodization was conducted under potentiostatic conditions using oxalic acid with a concentration of 0.2 M at 70 V anodization using a potentiostat (Keithley 2400 SM, Cleveland, OH, USA). Following the anodization, the oxide barrier layer was removed using 5 wt.% phosphoric acid at a temperature of 30 °C. Au, Pt, and AuPt alloys were deposited into the pores via electrochemical deposition from an aqueous solution of 8 mM HAuCl_4_·3H_2_O, 10 mM H_2_PtCl_6_, and mixtures of both with different mixing ratios. An electrochemical workstation (Princeton Potentiostat/Galvanostat Model 263A, AMETEK, Berwyn, PA, USA) was used for the deposition. Reference samples of Au and Pt NRs were fabricated as described above using 8mM HAuCl_4_·3H_2_O and 10 mM H_2_PtCl_6_, respectively. For comparison, thin films of Pt and AuPt alloys were processed by electrochemical deposition of 10 mM H_2_PtCl_6_ and different mixtures of 8 mM HAuCl_4_·3H_2_O and 10 mM H_2_PtCl_6_, respectively. The heat treatment was done in a tube furnace (Linn High Therm FRH, Bad Frankenhausen, Germany) at 400 °C. Electrodeposited Pt films were heated at temperatures up to 800 °C for times ranging from 1 to 2 h. A mixture of 10 mL deionized water, 5 mL ethanol, and 0.25 mL ammonium hydrogen difluoride was used for dissolving Si. The samples were characterized via scanning electron microscopy (SEM, Zeiss Ultra, Oberkochen, Germany), energy dispersive X-ray spectroscopy (EDX, INCA, Oxford, Oxford Instruments, Abingdon, UK), X-ray diffraction (XRD, X’Pert Pro diffractometer, PANalytical,, Kassel, Germany) in grazing incidence mode with a grazing angle of 2°, helium ion microscopy (HIM, Zeiss Orion NanoFab, Zeiss, Oberkochen, Germany), and transmission electron microscopy (JEOL 2100f at 200 kV, Akishima, Japan). Samples for TEM were prepared by focused ion beam sectioning.

## 3. Results

### 3.1. Pt Nanorods

**Figure 1:**
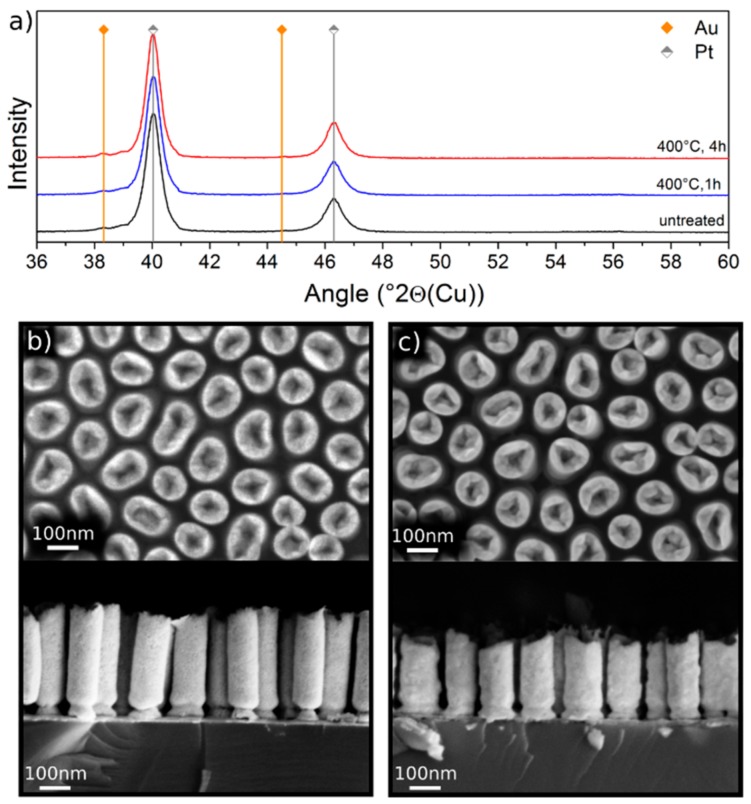
(**a**) XRD patterns of as-fabricated Pt nanorods (NRs) (black line), annealed at 400 °C for 1 h (blue line) and annealed at 400 °C for additional 3 h (red line). The peaks match the positions of the characteristic Pt peaks obtained from the reference sample (vertical grey lines). The 15 nm Au underlayer caused small peaks located at the characteristic 2θ positions of the Au peaks (vertical orange lines). (**b**) Top and side view backscattered electron (BSE) micrographs of Pt NRs before annealing, and (**c**) after annealing at 400 °C for 4 h. The NRs contracted after annealing and the Au underlayer was partially spheroidized.

The morphology of as-fabricated Pt NRs is displayed in Figure 1b. They are approximately 400 nm long and have a mean diameter of 80 nm. As can be seen, the Pt NRs consist of a solid base and a hollow tip. The behavior of these pure Pt NRs on annealing at 400 °C for different times was explored. When annealed at 400 °C for 1 h, no morphological or chemical changes could be detected. A longer annealing time of 4 h showed partial contraction of the NRs, probably as a result of diminishing porosity, and a break-up of the Au underlayer, which might be caused by AuSi intermixing or layer spheroidization, as evident in Figure 1c. The XRD patterns shown in Figure 1a confirm that annealing up to 4 h does not change the crystal structure of the NRs and no chemical change could be detected within the resolution of the Scanning Electron Microscope – Energy Dispersive Spectrometer (SEM-EDS). Peak positions of Pt (grey) and Au (orange) are marked in Figure 1a. The weak Au peak visible in Figure 1a is attributed to the 15 nm Au underlayer. After the heat treatments (blue and red lines), neither the formation of PtSi nor a shift of the peak positions was observed. These results are rather surprising, since PtSi formation in a SiAuPt thin film heterostructure was reported to occur at temperatures as low as 150 °C [7]. As will be shown later, the thin adhesion layer of Ti has no impeding effect on PtSi formation. Higher annealing temperatures to overcome the energy barrier and accelerate PtSi formation could not be investigated because they led to the collapse of the discrete Pt NRs. For this reason, we resorted to investigating corresponding thin film heterostructure samples. Samples of electrodeposited Pt film of thickness 40 nm on Si with the same sputtered Ti/Au/Ti layers as for the Pt NRs samples were used to study the effects of higher annealing temperatures and times on interfacial reactions.

The results are summarized in the supplementary information (Figure S1). Briefly a sequence of heat treatments between 400 °C and 800 °C for times ranging between 1 and 2 h did not result in the formation of any silicide phase. Instead interdiffusion between Au and Pt took place leading to the formation of an Au-rich (24 at% Pt) and a Pt-rich (83.7 at% Pt) alloy with compositions nearly reflecting the miscibility gap in the AuPt phase diagram, e.g., at 800 °C [27].

### 3.2. Au Nanorods

**Figure 2:**
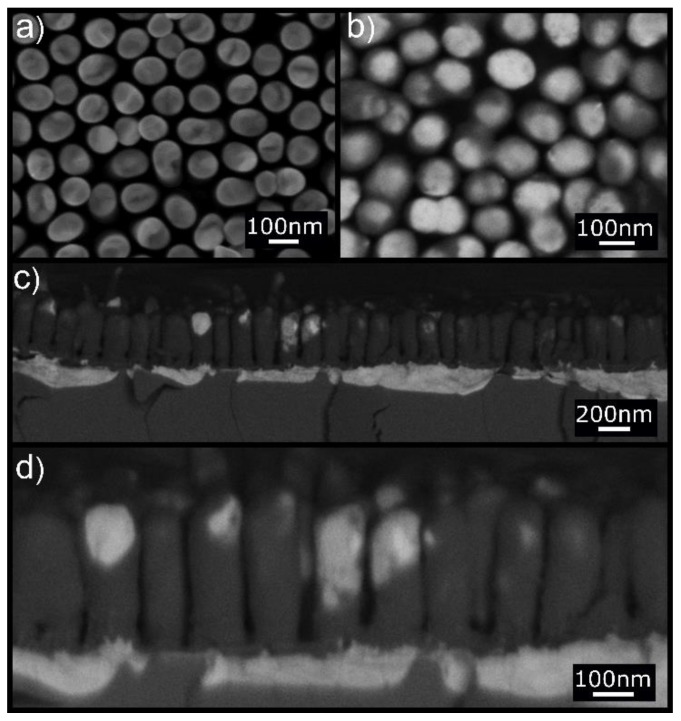
(**a**) Secondary electron (SE) SEM top view of the Au NRs before annealing. (**b**) BSE SEM top view of the Au NRs after annealing at 250 °C for 1 h. The high-resolution BSE image shows dark contrast in NRs with bright top caps. (**c**,**d**) BSE cross-section images at two different magnifications showing the NR structure after annealing. The same contrast characterizes the NRs and the silicon substrate. Some of the NRs are partially covered laterally with Au film. Notice also the presence of a thick Au layer (bright contrast) beneath the dark Si NRs.

The morphology of the as-fabricated Au NRs on substrates similar to that used for Pt NRs is shown in Figure 2a. They are approximately 320 nm long and 80 nm in diameter. Figure 2a shows the top view of the NR array where discrete NRs with homogenous chemistry can be observed. Annealing at 250 °C for 1 h resulted in substantial change in the NR chemistry as evident in the high-resolution backscattered electron (BSE) image of Figure 2b,d where dark contrast NRs with some bright contrast caps and regions are clearly visible. Such profound difference in BSE image contrast represents significant changes in chemical composition within the NRs.

We infer from subsequent investigations that the Au in the NRs was effectively replaced by Si transforming the Au NRs to Si NRs, and that some of the Au was displaced to the tips of the NRs to give a bright contrast. This is confirmed by in situ energy dispersive X-ray chemical analysis.

The cross-section images displayed in Figure 2c,d further show that a large amount of Au was diffused downward into the silicon substrate together with upward diffusion of Si into the NRs.

In contrast to the highly stable Pt NRs on Si discussed previously, the Au NRs were unstable on annealing exhibiting significant interdiffusion of Au and Si across the interface leading to effective replacement of Au by Si/SiO_2_ accommodated by Au diffusion down to the substrate. Note that such diffusion occurred at an annealing temperature far below that of the AuSi eutectic temperature. In this process, the Au NRs certainly promoted the formation of the Si NRs, not through a liquid phase, as for the VLS process, but simply via solid-state diffusion phenomena (we propose a mechanism for the Si NR formation in the discussion section).

### 3.3. AuPt Nanoalloy Nanorods

We now investigate AuPt nanoalloy NR arrays, fabricated similarly to the elemental Au and Pt NR arrays, to study how the presence of Pt in different concentrations will affect the diffusional transformation behavior observed in Au NRs on annealing. AuPt nanoalloy NRs with three different compositions were fabricated by electrodeposition in the AAO thin films. Their compositions and morphological peculiarities are listed in Table 1.

**Figure 3:**
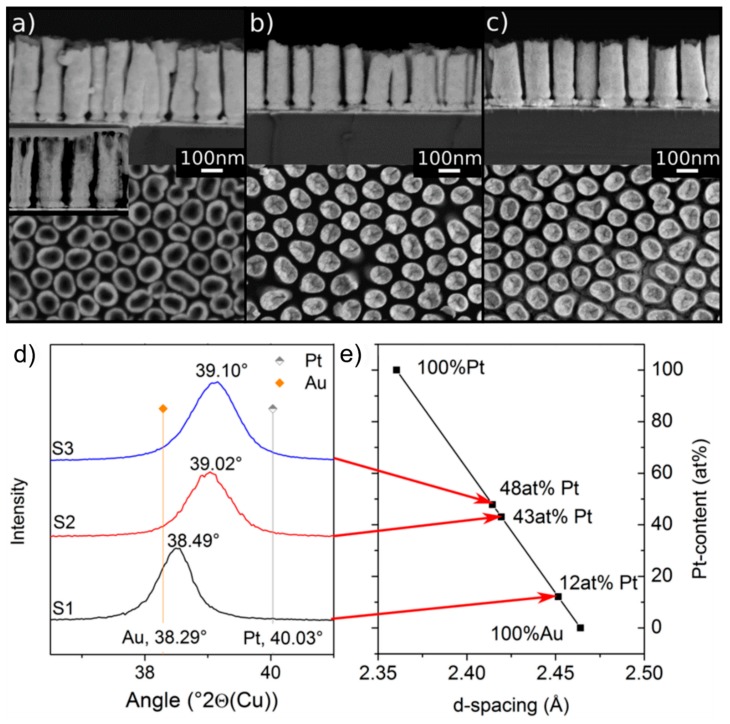
Side and top view SEM images of AuPt nanoalloy NR arrays with different Pt contents and different shapes. (**a**) Sample S1 (12 at% Pt): Partly hollow NRs, 350 nm length, the inset shows the Scanning Transmission Electron Microscope (STEM) image of the cross-section. (**b**) Sample S2 (43 at% Pt): Compact NRs, 280 nm long. (**c**) Sample S3 (48 at% Pt): Compact NRs, 280 nm long. (**d**) XRD spectra of the samples S1–S3, showing the 111 peaks located between the characteristic peak positions of Au and Pt. (**e**) Calculated Pt content of the three alloys using Vegard’s law.

The BSE micrographs of the samples in Figure 3 show a uniform chemical contrast within the NRs, confirming that they have a homogeneous chemistry. The sample with the lowest amount of Pt is partly hollow (Figure 3a) while the other samples are solid. Figure 3d shows the 111 peaks of all three samples before annealing. It is clear that the samples exhibit a single peak located between the characteristic peaks of elemental Au and Pt. The peak positions increased to higher 2θ values with increasing Pt content. From the peak positions, the Pt contents in each sample were determined using Vegard’s law, as indicated in Figure 3e.

**Figure 4:**
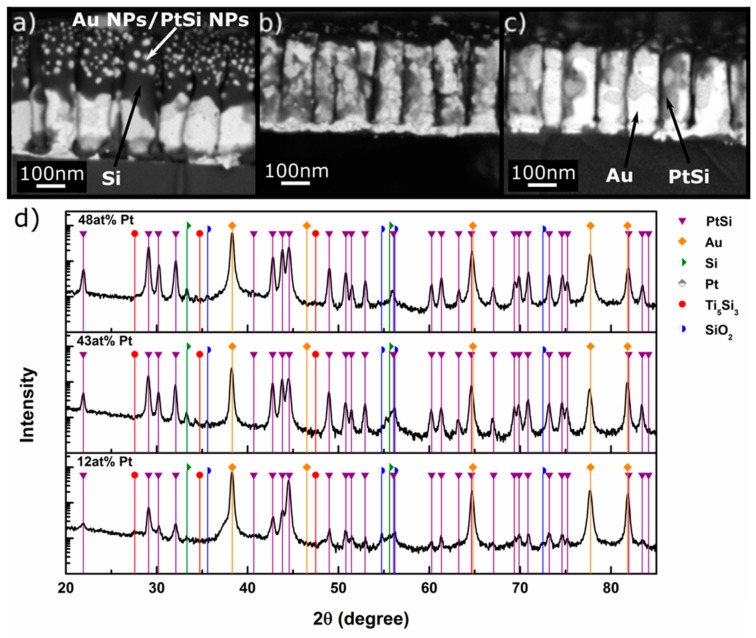
BSE micrographs of AuPt alloy NRs after annealing at 400 °C for 3 h. The brightest areas are Au, the second brightest PtSi, and the dark areas consist of Si or an AuSi alloy. The dark material on top of the NRs is probably pure Si. (**a**) Sample S1, (**b**) Sample S2, (**c**) Sample S3. (**d**) XRD patterns of NRs of AuPt alloy Sample S1 (bottom), Sample S2 (middle), and Sample S3 (top) after annealing for 3 h at 400 °C. PtSi peaks are marked in purple. The Ti adhesive layer led to the formation of Ti_5_Si_3_. All samples display PtSi peaks. The peaks (previously pertaining to the AuPt nanoalloys) that were located between pure Au and pure Pt positions shifted to the pure Au 2θ position.

The XRD patterns of the samples after annealing for 3 h at 400 °C shown in Figure 4d reveal the formation of new phases. Prominent among them is the rhombohedral intermetallic PtSi phase. It should be noted that Pt seems to have been fully consumed from the alloy to form PtSi since the 111 AuPt alloy peaks in Figure 3d shifted to the 2θ position of elemental Au. This confirms that the nanoalloy phase was no more present. As a corollary, the PtSi peak intensity increased with increase in the initial content of Pt in the AuPt NRs, signifying higher mass fractions of PtSi phase. There were also very weak peaks that could possibly be attributed to Ti_5_Si_3_, SiO_2,_ and Si, although we cannot be conclusive about their formation.

The microscopic investigations showed a broad range of features that are summarized below and attest to the complexity of the reactions the system undergoes. Taking a closer look at the BSE micrographs of the annealed samples in Figure 4, we distinguished different contrast shades that can be attributed to the different major phases identified in their XRD patterns. The phase with the brightest contrast is Au (the element with the highest atomic number in the heat-treated system). The phase with the second brightest contrast is most probably PtSi, and the darker appearing phase covering parts of the NRs is Si (or Si with a SiO_2_ scale). The tip of the NRs was always covered with the latter, and one can easily identify bright NPs within the NRs that are probably Au NPs (see below).

**Figure 5:**
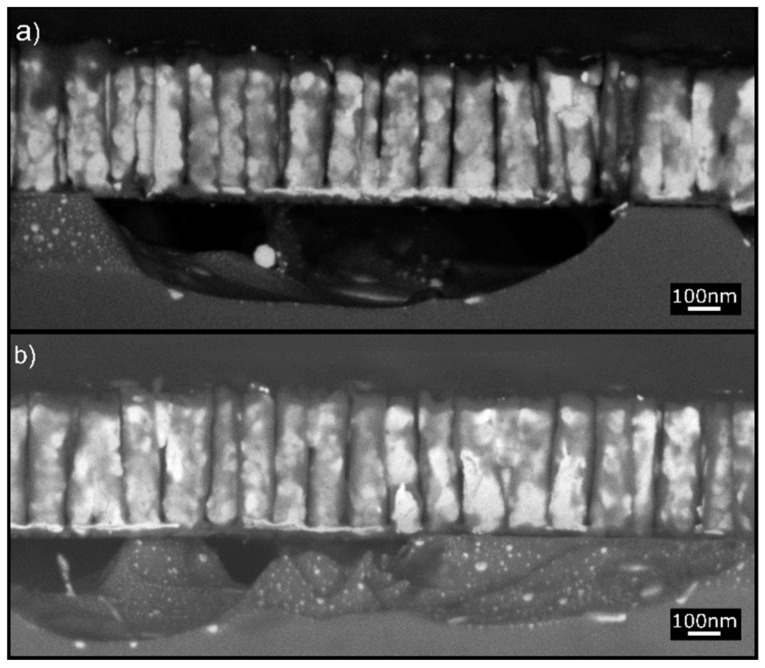
A closer look at Sample S2 reveals the size and chemistry of the intermixing zone below the NRs. (**a**) The 250-nm-deep AuSi alloy intermixing zone with embedded Au particles and cavities. (**b**) V-shaped cavities in the intermixing zone with Au NPs (bright spots).

Directing now our attention to the substrate beneath the NRs, which formerly consisted of Si covered with an Au film, we can distinguish between different intermixing zones, particularly for the Pt-rich NRs, as exemplified in Figure 5 for sample S2 (43 at% Pt). The salient features are: (1) The formation of a Kirkendall void zone by vacancy coalescence in the Si substrate, (2) and the presence of Au precipitates in the Si substrate matrix. These observations are consistent with the phenomenon of interdiffusion of Au and Si at Au/Si junctions described by Cros and Muret [1]. According to them, the orientation of Si crystal has a strong influence on the interdiffusion between Au and Si. In our samples, the Kirkendall cavities had the characteristic V-shaped grooves they describe for the 100 silicon reacting with gold. The V shape is ascribed to the slower dissolution of Si atoms in the 111 direction. They describe the whole process in terms of the formation of metastable gold silicides at temperatures around 200 °C, followed by a phase separation of Au monocrystals in an AuSi alloy at higher temperatures of around 400 °C. In our samples, we can clearly see Au agglomerates up to 300 nm deep into the substrate. This means that intermixing must have occurred in this area as well as in the NRs.

The NRs consisted after annealing of NPs of PtSi and Au surrounded by Si, and possibly a surface layer of Si/SiO_2_. The cavities were caused by the faster upward diffusion of Si into the NRs compared to the downward diffusion of Au. Migrating Si atoms readily reacted with Pt atoms to form the silicide. Consequently, Pt atoms progressively exited the nanoalloy solid solution form leaving elemental Au atoms to accumulate. Si diffusion was accelerated by the presence of Au atoms rejected during PtSi formation. Since Si is known to diffuse to the surface and oxidize in Au/Si junctions (see also Figure S3, supplementary online information) [5], the material with dark contrast at the very tip of the NRs was most likely Si covered by a thin SiO_2_ scale. It is notable that the samples with the highest initial amount of Pt showed the least amount of Si/SiO_2_ in Figure 4. This could be attributed to the formation of PtSi, which captured and bound the Si flux, eventually hindering their migration to the tip of NRs. As long as free Pt atoms were available in the nanoalloy for reaction with Si, they acted somewhat as a diffusion barrier. After all Pt atoms were consumed to form PtSi, Si atoms could migrate to the tip of the NRs. This happened when the Pt content of the initial AuPt alloy was low.

**Figure 6:**
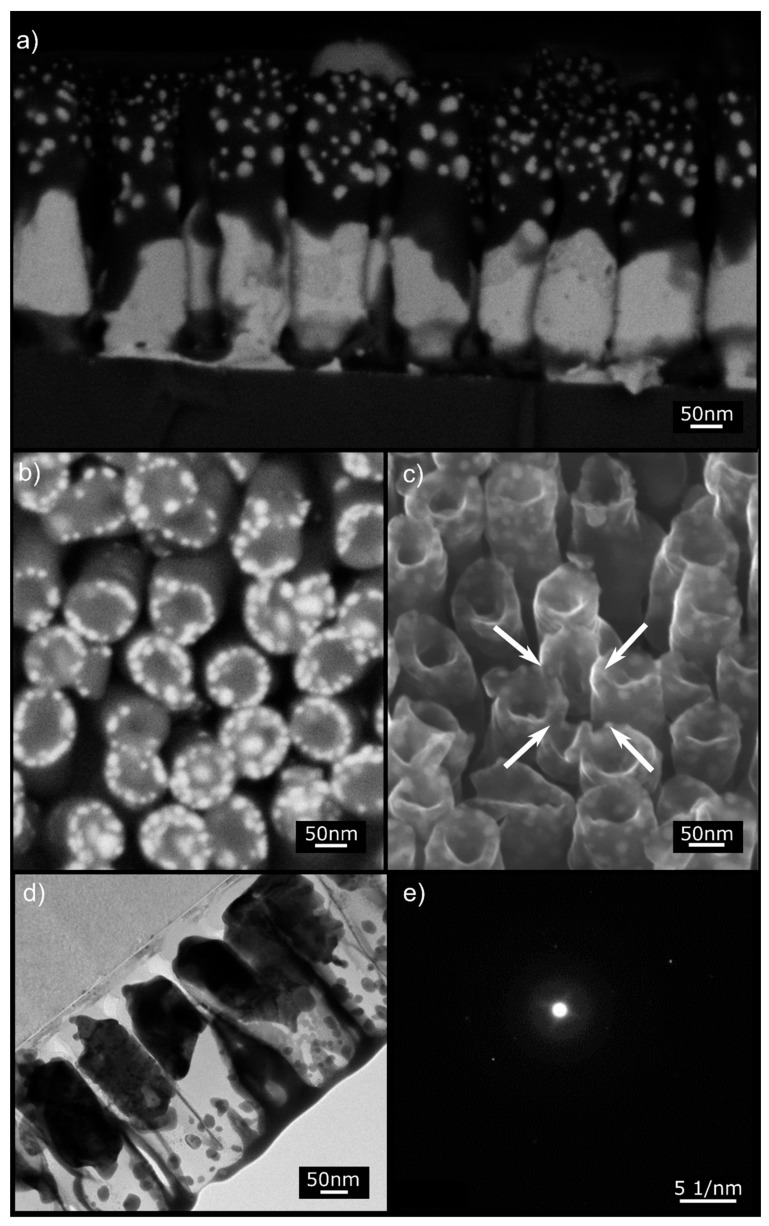
Electron microscopy images of Sample S1. (**a**) SEM BSE of the cross-section. (**b**) Top view SEM BSE micrograph showing the NRs tips displaying crowns of embedded NPs. (**c**) Helium ion microscope (HIM) SE image showing the hollow tip morphology of the NRs after annealing. The NR in the middle (arrows) is (on purpose) nanomachined at a specific angle to reveal that Au NPs are embedded in the Si matrix. (**d**) STEM image of a NR lamella showing the Au NPs embedded in the amorphous Si matrix (see the selected area diffraction pattern (**e**)). The film at the top of the NRs is the tungsten film that serves as a support for the lamella during focused ion beam (FIB) machining.

Now we focus on the Au12Pt NR sample, because it afforded some interesting microstructural features, namely Au and PtSi NPs that seemed to be embedded in a Si matrix in the NR structure. The top view micrograph of Figure 6b shows that the NRs displayed crowns of NPs. The helium ion microscope (HIM) image in Figure 6c (Figure S2), unraveled an additional detail, namely that the NPs were literally embedded in the Si/SiO_2_ phase. STEM and TEM investigations of this sample after annealing confirmed the amorphous nature of the Si/SiO_2_ envelop, displayed in Figure 6d. More analysis of the embedded NPs revealed their multiphase character.

For instance, selected area electron diffraction pattern (SAED) shows that the large particles at the base of the NRs consisted of a mixture of two different nanophases, namely Au and PtSi, as in the example of Figure S4a. The smaller particles that formed away from the NR base required nanobeam diffraction as they were too small for conventional SAED. They, too, were largely composed of a mixture of Au and PtSi nanophases, as demonstrated in the EDS spectrum of Figure S4c. It was difficult to obtain an easily discernible composite diffraction pattern in this region as it was laborious to find a good zone axis for both nanophases. Selected area EDS results could be obtained for the larger nanoparticles near the base of the NRs, which also confirmed the presence of Au and Pt in this region. Based on the XRD and TEM investigations above, we ascertained the presence of Au and PtSi NPs that were embedded in amorphous Si/SiO_2_ NRs.

## 4. Discussion

The results presented above show that diffusion phenomena at the interface between Au/AuPt nanoalloy NRs and the Si substrate led to the formation of specific nanophases whose distribution and amount were intimately dependent on the composition of the as-grown NRs. Pure Pt NRs, however, remained unchanged at the maximum annealing temperature and time investigated here. This result is, at first glance, in contradiction with what was published so far on sputtered Pt layers on Si [5,6]. However, we should bear in mind that sputtering, as a highly energetic process, leads to intermixing of Pt with the underlying material [6]. The resulting metastable system is readily reactive, and Pt silicides form at rather low annealing temperatures. This contrasts with electrodeposition, which leads to distinctly separate layers at room temperature. Our results on the absence of any silicide phase after annealing of an electrodeposited Pt layer on Si/Ti/Au even at temperatures as high as 800 °C, Figure S1 (supplementary information), confirmed that intermixing is a prerequisite for Pt silicide formation. In order to further bolster this, a 5 nm Pt layer was sputtered on the same heterostructure as above. The XRD patterns obtained in the sputtered, non-annealed state unambiguously showed that the resulting layer was, in fact, a metastable AuPt nanoalloy, as the 111 peaks were between those of pure Au and Pt (Figure S5, supplementary information). Annealing this system readily led to the formation of PtSi with the Au 111 peak shifting to its characteristic position. In contrast to electrodeposited pure Pt, the Au atoms in the pure Au and AuPt nanoalloys readily diffused out of the NRs, allowing Si from the substrate to diffuse into the NRs. Hence, this is a possible way to fabricate Si NR arrays starting from Au NRs on Si substrate. This occurred by solid state diffusion at temperatures much lower than the AuSi eutectic temperature. This is characteristically different to the VLS method of growing Si NRs where the AuSi eutectic alloy liquid droplet is formed at the tip, allowing Si atoms to be continuously added to the NR from the eutectic liquid. In the VLS method, the AuSi eutectic alloy finally solidified at the tip when the system was cooled to room temperature. Such eutectic alloy droplet was not formed in our way of fabricating Si NRs. Besides, there is no gaseous phase involved as in the VLS method to replenish Si atoms. Si diffused into the NR from the substrate.

Interdiffusion between Au and Si is well studied as it is relevant to gold wire bonding and ohmic contacts in microelectronics. It is thought that Si–Si bonds are weakened in the presence of Au atoms permitting fast diffusion of Si into Au. In many thin film studies, Si from substrate was shown to reach the surface through the Au film and get oxidized, which prompted researchers to describe the surface as a “chemical potential sink”. The driving force for diffusion of Si to the surface was most probably related to much lower surface chemical potential than that of Au. Buttner et al. [28] used a method resembling VLS technology to axially convert Si NR grown by molecular bean epitaxy to NRs of SiO_2_ at 850 °C using Au droplet as a catalyst at the NR tips. The transformation rate of Si to SiO_2_ was much faster than conventional diffusional oxidation from the surfaces. They found that SiO_2_ formed above the Au droplet gradually pushed the droplet down towards the substrate as SiO_2_ conversion progressed. The diffusion path for the Si atoms is then from the NR through Au droplet to the surface for oxidation, again echoing the role of surface as a chemical potential sink. This mechanism occurs even at low temperatures, below the eutectic temperature (at 250 °C) of the AuSi system if the oxidation reaction of Si could be accelerated by having a wet environment. This experiment of converting Si NRs into SiO_2_ NRs is similar to our situation described in this paper but the environment was not oxidizing. Therefore, Si atoms that reached surface chemical potential sink remained largely unoxidized (a SiO2 thin film might, however, form on the surface). In consequence, Au atoms were forced out of the NRs into the substrate possibly assisted by internal stresses caused by diffusion. The result was a confined axial diffusion and growth of Si, which adopted the pristine morphology of the Au NRs. The Si flux was higher than the Au flux, hence, the formation of Kirkendall voids in the substrate near the NR interfaces.

The existence of Au film at the tip of the NRs in some cases may have been due to some barrier to Au diffusion. In a designed experiment, Buttner et al. deliberately deposited a thin SiO_2_ layer at the Si NR tip before depositing Au [23]. They showed that Au and Si could not diffuse through this oxide layer and thus the SiO_2_ NR formation was arrested. In effect, Au ceased to act as a catalyst because of this diffusion barrier. Similarly, the formation of an oxide scale on the Si NRs may have prevented some of the Au from diffusing out of the NR in our case.

With respect to AuPt nanoalloys, a similar mechanism to pure Au may be proposed. It should first be noted that the AuPt bulk system shows a miscibility gap below 1620 °C with the formation of two face centred cubic (fcc) phases, an Au-rich phase at the Au side and a Pt-rich phase at the Pt side [27]. We may then state that the AuPt nanoalloys with high Pt concentrations were metastable, and should lead to phase separation upon heat treatment. This was shown by Braidy et al. [29], who studied the phase separation in AuPt nanoparticles upon heat treatment at temperatures between 300 and 800 °C. Their results showed that the concentration of Au in the NPs was larger than predicted by the phase diagram for bulk material [27]. Further, Pt reacted readily with Si to form stable (and metastable) Pt silicides with different stoichiometry and structure [17], among them was the equiatomic PtSi compound with a rhombohedral structure (space group Pnma). We have shown above that this compound preferentially forms in our system.

The composition and phase contents in the final nanophase NRs obtained in AuPt alloy systems could be manipulated by varying the initial alloy composition and the annealing conditions.

It was then straightforward to interpret the microscopic images invoking Si diffusion towards the AuPt NRs and solid-state reaction with Pt. The formation of PtSi continued until all Pt was consumed while Au was rejected at the interface. The formation of PtSi was, therefore, concomitant with Si diffusion towards the NR tip (and Au diffusion downward) as described above for pure Au. This resulted in composite NRs with PtSi, Au, and Si/SiO_2_ phases. The NP crowns depicted in Figure 6b likely formed as the Si front advanced, similarly to the case described above for pure Au NRs. Spheroidization probably occurred because of the higher annealing temperature of the AuPt NRs.

**Figure 7:**
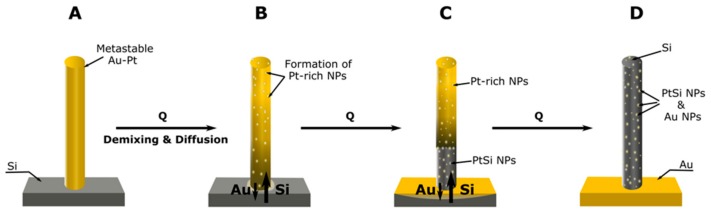
A schematic of the mechanisms proposed to govern the formation of Si/SiO_2_ NRs via upward axial solid-state diffusion of Si and downward Au diffusion towards the substrate. When pure Au NRs on Si substrate were annealed, only Si/SiO_2_ NRs with residual Au were formed. When AuPt on Si were annealed, discrete phases, mainly PtSi and Au NPs, formed in Si/SiO_2_ envelop. (**A**) Depicts an as-grown AuPt NR. (**B**) On annealing, Pt-rich particles formed in Au-rich matrix and concomitant interdiffusion. (**C**) Reaction proceeds with the formation of intermetallic PtSi and Au diffusion into Si. (**D**) Reaction products with the formation of Si/SiO_2_ NR that contained PtSi NPs and Au NPs from Au that did not diffuse into the Si substrate.

A schematic summary of the proposed mechanism for both Au and AuPt NRs is given in Figure 7. When pure Pt NRs were on Si substrate, such accelerated diffusion of Si to the NR tip did not occur possibly because Pt did not weaken the SiSi bonds as in the case of Au. Therefore, any reaction was confined to the interface, as with most other interface reactions. This would be a very slow process and, hence, was not detected at most temperatures except at high temperatures in our experiments.

## 5. Conclusions

In this work we shed light on the complex interactions between Si substrate and Au, Pt, and AuPt nanoalloys in NR forms. We showed that in pure Pt NRs and Pt thin films on Si substrates no interdiffusion or interreactions were observed until annealing conditions as high as 800 °C for 4 h were reached. Hence, the Pt NRs were very stable when they were produced in Si substrates.

In the case of pure Au NRs on Si substrates, however, ready interdiffusion was observed even at annealing temperatures as low as 150 °C, resulting in the transformation of the Au NRs to Si NRs. Substantial diffusion of Au into the substrate was also evident and Kirkendall voids were detected in the substrate. Hence, Au NRs on Si substrates were very unstable. This could be attributed to the accelerated diffusion of Si through Au towards the NR surface, as commonly seen in VLS method of producing Si/SiO_2_ NRs. This could be a viable method of producing Si nanorods on Si substrates at low temperatures by solid-state diffusion without involving any vapor phase as in the VLS method.

In the case of AuPt nanoalloy NRs on Si substrate, the interactions were complex on annealing. Significant diffusion of Si into the NRs was noted, leading to the formation of PtSi besides the presence of elemental phases Au, Pt, and Si after annealing at 400 °C for 3 h. The relative amounts of the phases depended on the Pt content of the initial nanoalloy. For low initial Pt content (12 at%) no elemental Pt was detected after annealing; all the Pt was converted to PtSi. For the higher Pt content nanoalloys, some remnant elemental Pt remained along with PtSi after annealing, indicating its incomplete consumption for the silicide formation reaction. The remnant Pt prevented Si diffusion to the tip of the NR, as observed in the case of pure Au NRs, as Pt atoms captured the Si flux and bound them in the PtSi phase. Only after all Pt atoms in the nanoalloy NRs were consumed to form PtSi, Si diffusion through the alloy could occur towards the NR tip.

This study shows that the presence of Au with Pt strongly promotes the formation of PtSi, which could be attributed to the accelerated, confined axial diffusion of Si into the Au matrix of the alloy NRs. As a corollary, once all Au atoms have diffused out into substrate, the Si flux should cease, effectively ending the consequent reaction with Pt. Therefore, the Pt atoms do not play an active role in the interdiffusion process but readily capture the diffusing Si atoms to form the silicide phase leading to a mixed nanophase nanorods, which might have beneficial properties since the structure consists of heterojunctions of two semiconducting phases, Si and PtSi.

Potential applications of AuPt nanoalloys are mainly in (electro)catalysis. Plasmonics could also be an interesting application field as the presence of Pt blue shifts the absorption peak of Au for specific Pt concentrations (to be published). Heat treatment of Pt-lean AuPt alloys results in the formation of Au NPs in a Si/SiO_2_ envelop that should protect them against external environments, which could also be interesting for plasmonic applications.

## Figures and Tables

**Table 1:** AuPt nanoalloy NRs. Dimensions and compositions. The cluster size was calculated from the XRD data using the Scherer formula.

Sample	NR Diameter (nm)	NR Length (nm)	Pt-Content (at%)	Cluster Size (nm)	
				Before Annealing	After Annealing
S1	100	350	12	16.1	37.1
S2	90	280	43	15.2	35.4
S3	90	280	48	14.2	36.5

## Supplementary Materials

The following are available online at https://www.mdpi.com/2079-4991/10/1/68/s1, Figure S1: electrodeposited 40 nm Pt layer before and after annealing, Figure S2: helium ion microscope (HIM) image of sample S1. Figure S3: SEM cross-section of a disrupted Au layer. Figure S4: TEM images, selected area electron diffraction patterns, and EDS analysis for Au12Pt NR sample. Figure S5: SEM image and XRD patterns of a sputtered Pt-layer on Si/Ti/Au heterostructure, before and after annealing.
