# Supplementary material for: Formation of Si Nanorods and Discrete Nanophases by Axial Diffusion of Si from Substrate into Au and AuPt Nanoalloy Nanorods
Source: Nanomaterials (Basel). 2019 Dec 27;10(1):68. doi: 10.3390/nano10010068 (PMC7022470; doi:10.3390/nano10010068)
Supplement: Supplementary file 1 [file nanomaterials-10-00068-s001.pdf]

nanomaterials

Supplementary Material

Formation of Si Nanorods and Discrete Nanophases by Axial Diffusion of Si from Substrate into Au and AuPt Nanoalloy Nanorods

Nele Berger [1], Ayoub Laghrissi [1], Yee Yan Tay [2], Thirumany Sritharan [2,*], Jacek Fiutowski [3], Horst-Günter Rubahn [3] and Mohammed Es-Souni [1,*]

[1] Institute for Materials & Surface Technology, Kiel University of Applied Sciences, 24149 Kiel, Germany; Nele.berger@gmx.net (N.B.); Ayoub.laghrissi@fh-kiel.de (A.L.)

[2] School of Materials Science and Engineering, Nanyang Technological University, Singapore 639798, Singapore; yytay@ntu.edu.sg (Y.T.); assritharan@ntu.edu.sg (T.S.)

[3] Mads Clausen Institute, University of Southern Denmark, NanoSYD, Alsion 2, 6400 Sønderborg, Denmark;

* Correspondence: mohammed.es-souni@fh-kiel.de

Received: 13 November 2019; Accepted: 24 December 2019; Published: 27 December 2019

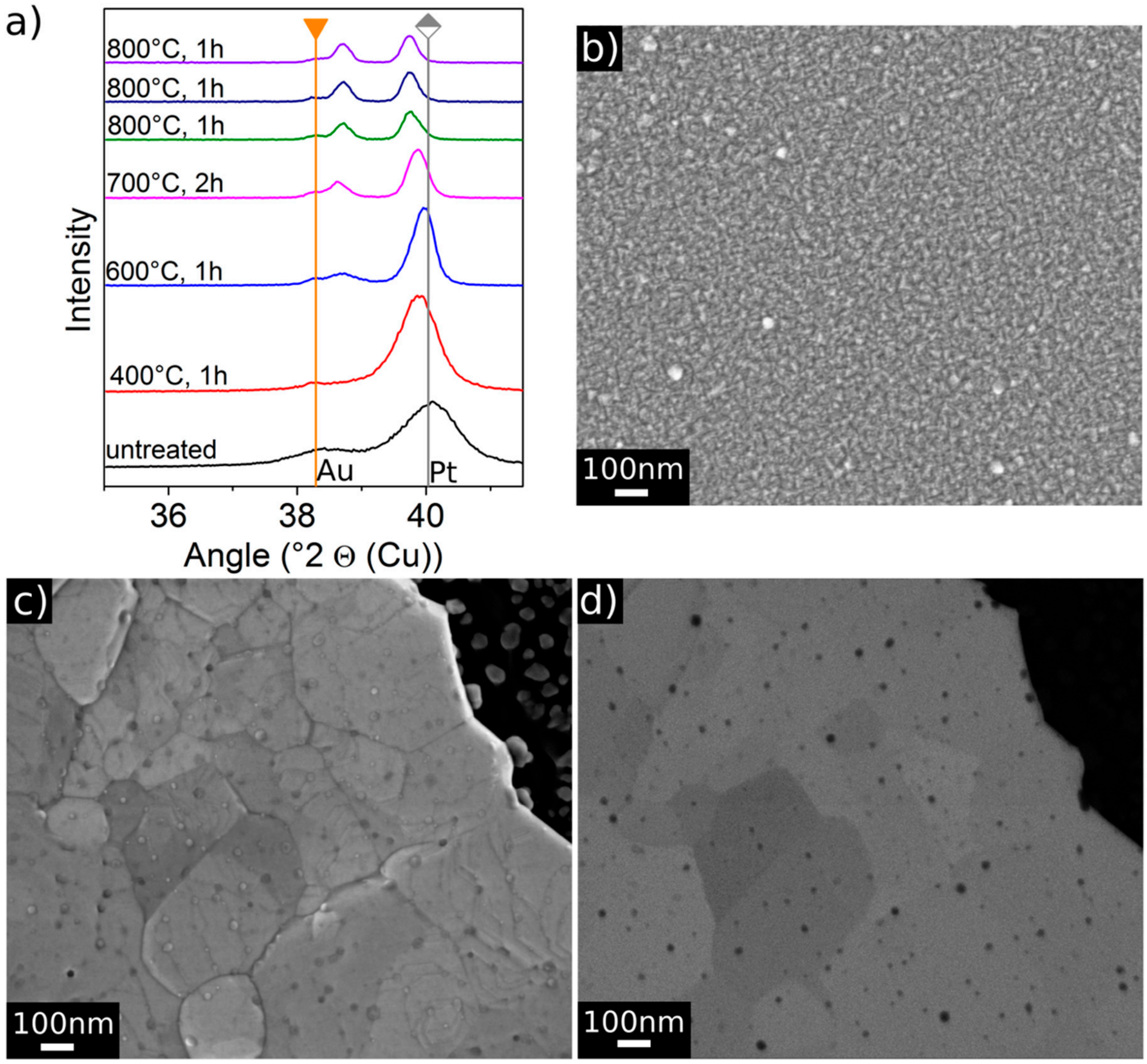

Figure S1. (**a**) XRD patterns of a 40 nm Pt layer electrodeposited on a Ti(6 nm)/Au(15 nm)/Ti(2 nm) heterostructure on a Si (100) substrate before annealing (black line) and after annealing between 400 °C and 800 °C for the times indicated. Before annealing the peaks match the characteristic peaks of Au and Pt. After 1 h the Pt peak shifts towards the Au peak while the Au peak decreases in intensity implying the formation of a Pt-rich Au-Pt alloy. After 1 h at 600 °C a third peak appears between the Au and Pt peak positions showing the formation of another, Au-rich, Au-Pt alloy. The annealing steps at 600 °C and 700 °C lead to an intensification of the peak of the Au-rich alloy while the Au-peak is still visible and the peak of the Pt-rich alloy loses intensity. After annealing at 800 °C, the peak positions do not change anymore and the structure consists of 3 phases: Au, Au-rich Au-Pt alloy (24 at% Pt) and Pt-rich Au-Pt alloy (83.7 at% Pt). (**b**) SEM image of the structure before annealing. (**c**) SEM image of the sample after all annealing steps showing a coarse grained structure embedded with small particles. (**d**) The backscatter micrograph shows the chemical contrast of the different phases. The darker contrast of the small particles suggests that they might consist of Si.

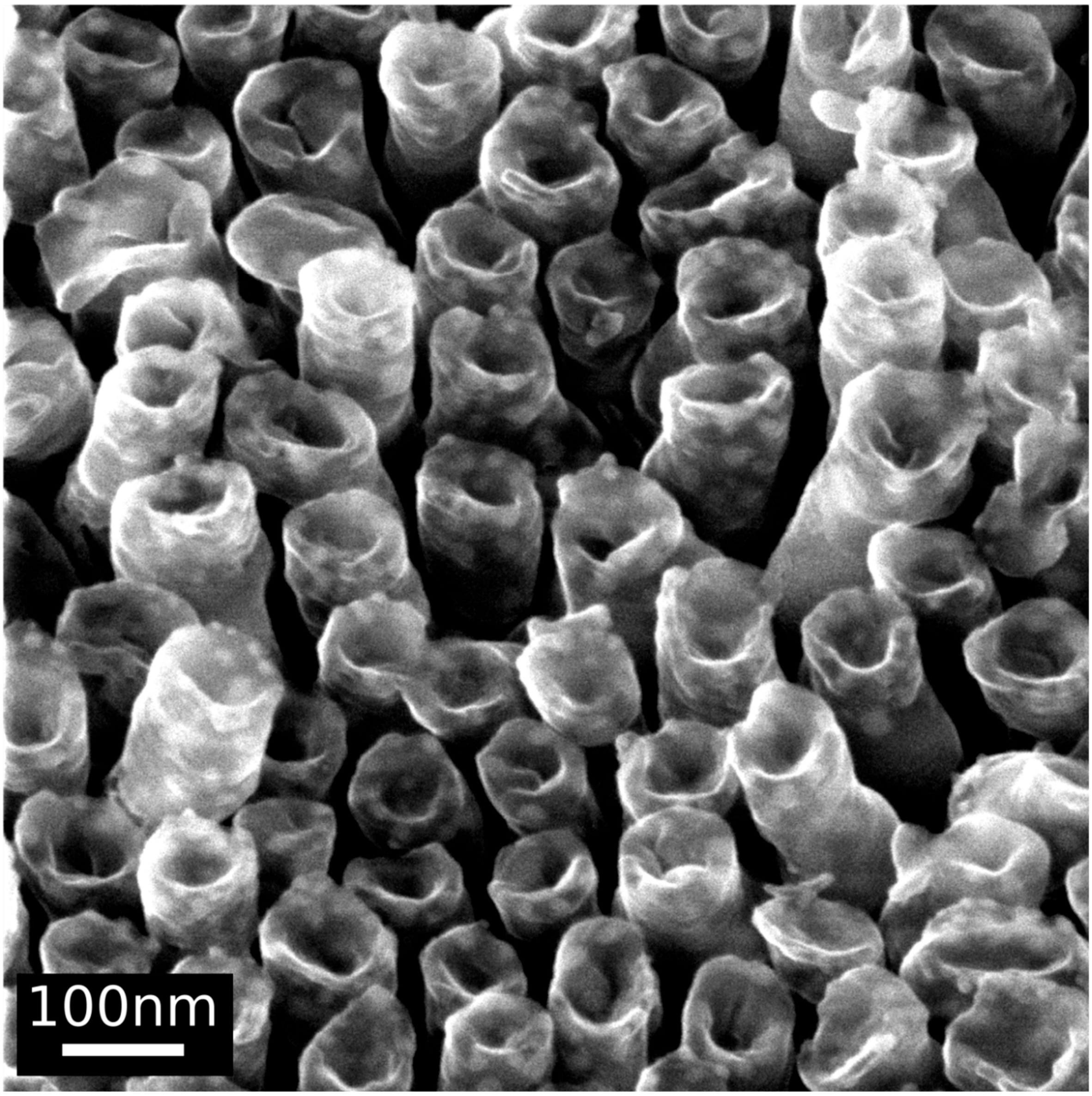

Figure S2. HIM image of sample S1 showing that Au particles near the tip are embedded in a matrix of Si or Si-rich Au-Si alloy.

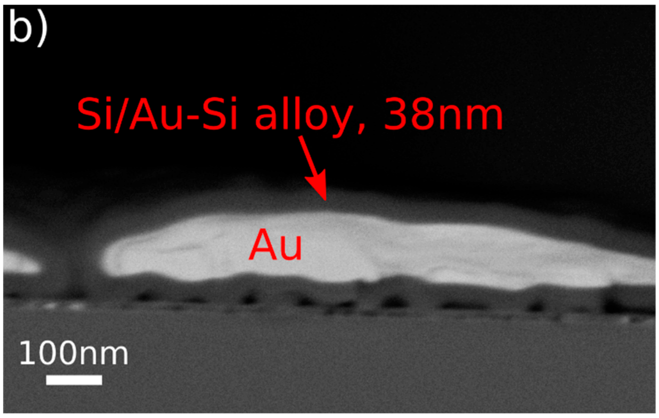

Figure S3. Backscatter micrograph of cross section of disrupted Au-layer, electrodeposited on Ti/Au/Ti heterostructure and Si (100) substrate after annealing at 400 °C for 3 h. The core consisting of Au is covered by a 38 nm layer of Si/SiO_2 or Si-rich Au-Si alloy/SiO_2.

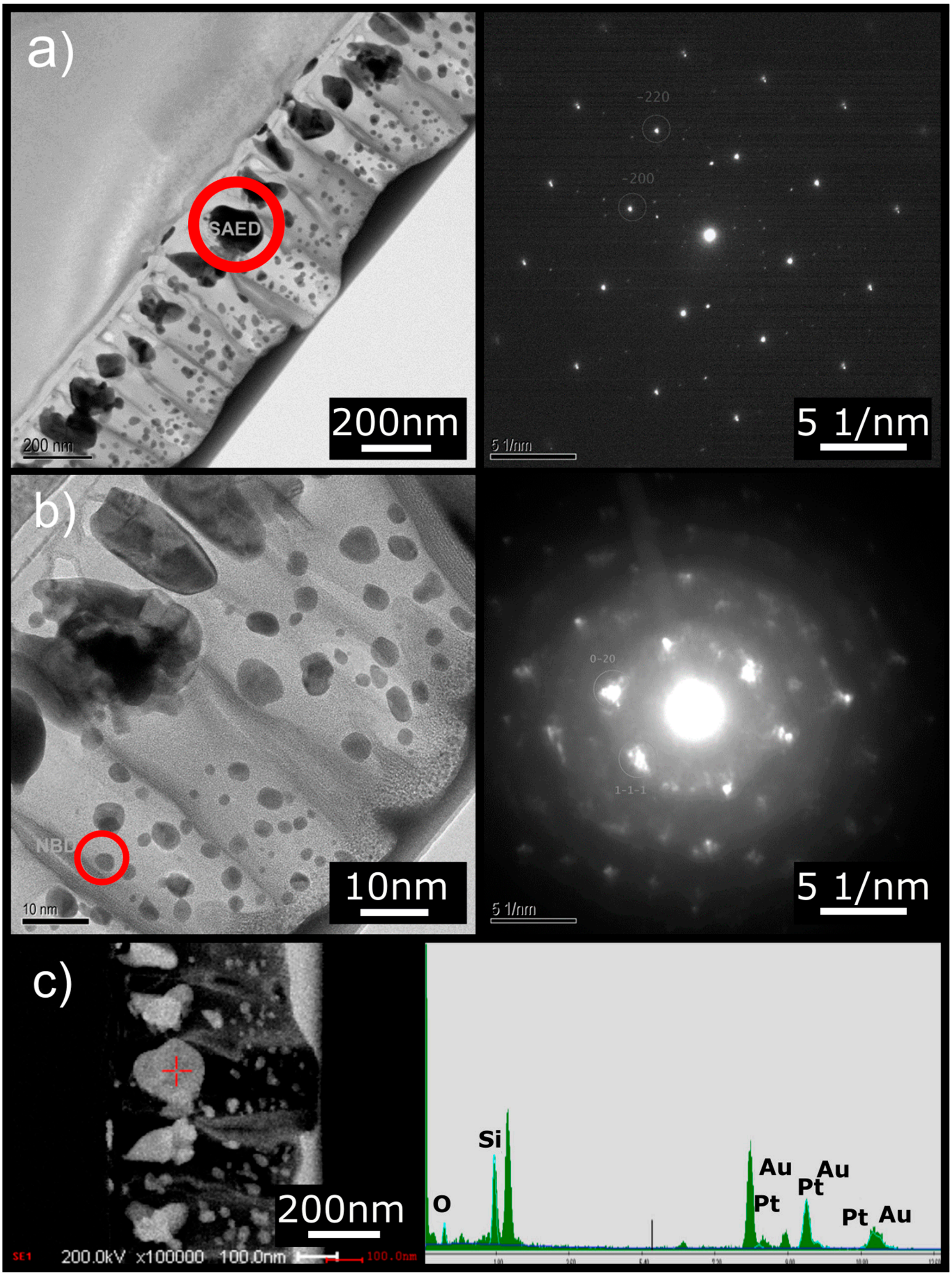

Figure S4. TEM bright field images and diffraction patterns (**a**, **b**) of the annealed Au-12wt%Pt NRs showing PtSi and Au nano particles in Si matrix. The selected area electron diffratction patterns of the circled areas. (**c**) STEM micrograph and the corresponding energy dispersive spectrographic (EDS) results of the big particle at the base of the NR (shown by red +). The un-indexed peak correponds to tungsten that supports the FIB sample sectioning.

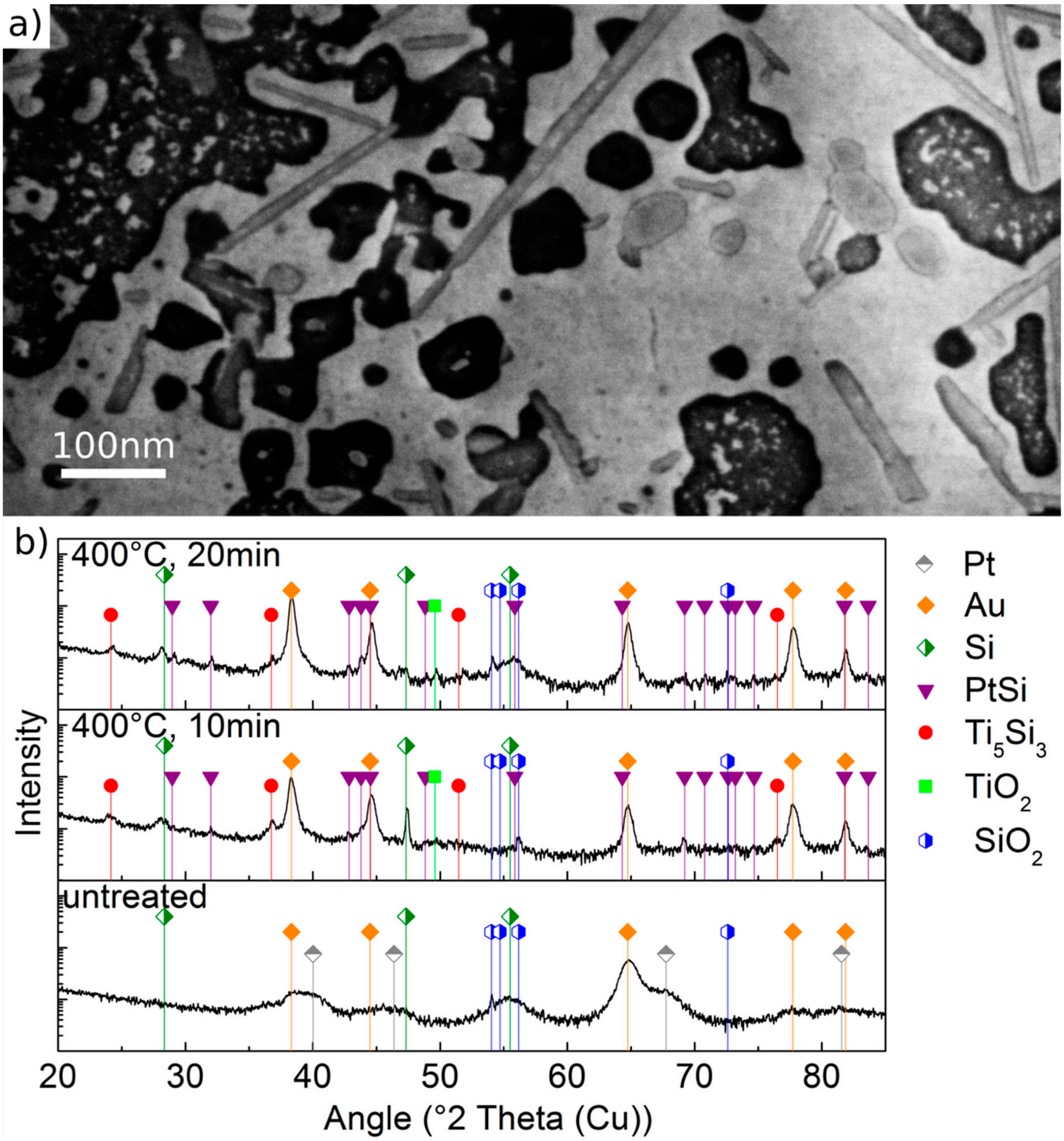

Figure S5. (**a**) SEM backscatter micrograph of a 5nm Pt layer sputtered on a Ti(6nm)/Au(15nm) heterostructure on a silicon (100) substrate after heating at 400°C for 20min. PtSi and Ti_5Si_3 have a darker contrast than the surrounding Au. The needle-shaped structures consist of Ti_5Si_3. The Au-layer is partly disrupted and the substrate can be seen under it (anthracite/black shade). There are also dark spots on top of the Au-layer, which we interpret as Si or a Si-rich phase. (**b**) XRD pattern of the sample before heating (bottom), after heating at 400°C for 10min (middle) and after heating at 400°C for 20min (top). Before heating only the peaks caused by Au, Pt and Si are visible. The peaks of Au and Pt are merged to one wide peak before heating. After 10 min of heating, the Au-peak is sharper and more pronounced, the initial Si-peaks have decreased and new Si-peaks and peaks of PtSi, SiO_2 and Ti_5Si_3 occur additionally. After 20min, the initial Si-peaks have increased again, the newly formed Si-peaks have slightly decreased and new SiO_2-peacs have occurred.
